# Supplementary material for: Class-weighted Dempster–Shafer in dual-level fusion for multimodal fake real estate listings detection
Source: PeerJ Comput Sci. 2025 May 27;11:e2797. doi: 10.7717/peerj-cs.2797 (PMC12190670; doi:10.7717/peerj-cs.2797)
Supplement: Supplemental Information 3 [file peerj-cs-11-2797-s003.docx]

**Steps to Execute Feature and Decision Fusion Codes**

1. In <https://github.com/maifuza/property-listings>, there are two Python scripts and a dataset file in Excel format.
2. Download the scripts and the dataset into a new folder.
3. Run the code using Jupyter in Anaconda Navigator.
4. Execute the features fusion.ipynb for feature fusion and decision fusion.ipynb for decision fusion.
